# The awareness of patients with non - muscle invasive bladder cancer regarding the importance of smoking cessation and their access to smoking cessation programs

**DOI:** 10.1590/S1677-5538.IBJU.2016.0014

**Published:** 2017

**Authors:** Emrah Yuruk, Murat Tuken, Aykut Colakerol, Ege Can Serefoglu

**Affiliations:** 1 Department of Urology, Bagcilar Training and Research Hospital, Istanbul, Turkey

**Keywords:** Urinary Bladder Neoplasms, Patients, Epidemiologic Studies

## Abstract

**Objectives:**

Smoking is the most important risk factor for bladder cancer and smoking cessation is associated with reduced risk of tumor recurrence and progression. The aim of this study is to assess the awareness of non-muscle invasive bladder cancer (NMIBC) patients regarding the importance of smoking cessation, determine their access to smoking cessation programs and the effects of smoking cessation on recurrence rates of NMIBC.

**Materials and Methods:**

NMIBC patients who were followed with cystoscopy were included in the study. Their demographic properties were recorded, along with their smoking habits, awareness regarding the effects of smoking on bladder cancer and previous attempts for smoking cessation. Moreover, the patients were asked whether they applied for a smoking cessation program. Recurrence of bladder cancer during the follow-up period was also noted.

**Results:**

A total of 187 patients were included in the study. The mean age was 64.68±12.05 (range: 15-90) and the male to female ratio was 167/20. At the time of diagnosis, 114 patients (61.0%) were active smokers, 35 patients (18.7%) were ex-smokers and 38 patients (20.3%) had never smoked before. After the diagnosis, 83.3% of the actively smoking patients were advised to quit smoking and 57.9% of them quit smoking. At the time of the study, 46.52% of the NMIBC patients were aware of the link between smoking and bladder cancer, whereas only 4.1% of the smoking patients were referred to smoking cessation programs. After a mean follow-up of 32.28±11.42 months, 84 patients (44.91%) had recurrence; however, current smoking status or awareness of the causative role of smoking on NMIBC did not affect the recurrence.

**Conclusion:**

In our study group, the majority of the NMIBC patients were not aware of the association between smoking and bladder cancer. Although most of the physicians advised patients to quit smoking, a significant amount of the patients were still active smokers during follow-up. Only a small proportion of patients were referred to smoking cessation programs. Urologists should take a more active role in the battle against smoking and refer those patients to smoking cessation programs. Larger study populations with longer follow-up periods are needed to better demonstrate the beneficial effects of smoking cessation on recurrence rates.

## INTRODUCTION

Based on the data from International Agency for Research on Cancer, bladder cancer is the 6^th^ most commonly diagnosed cancer of males and the 9^th^ leading cause of cancer deaths worldwide, with an estimated 330.400 new cases and 123.100 deaths for 2012 (1). Moreover, with €2.9 billion health care costs, bladder cancer is accounted for 3% of all cancer costs in the European Union in 2012 (2).

Epidemiologic studies clearly show that cigarette smoking is strongly associated with an increased risk of bladder cancer (3-6). Moreover, smoking increases the risk of bladder cancer as the second smoking-associated cancers among survivors of kidney, head and neck, and stage I lung cancers (7). In a case control study, Howe et al. noted that elimination of smoking from the entire population would result in a 61% decrease of bladder cancer cases in men and 26% in women (8). Furthermore, a large population based bladder cancer study revealed that the risk of having a bladder cancer was significantly higher (OR=3.08; CI 95%, 1.16-8.22) among female lifelong nonsmokers exposed to tobacco smoke at home during childhood (9).

Despite the establishment of public awareness regarding the association between smoking and cancer along with the vigorous efforts of global or local smoking cessation organizations, no significant changes in prevalence or cessation of smoking among Medicare Advantage participants between 2005 and 2012 were observed (10).

Patients with non-muscle invasive bladder cancer (NMIBC) are under great risk of recurrence and progression and thus require close surveillance programs. It has been documented that patients with bladder cancer made more than half a million outpatient visits to urology clinics in 2000 (11). Despite this invaluable opportunity to inform patients regarding the importance of cessation of smoking, the traditional role of urologists in treatment of bladder cancer is mainly toward treatment rather than prevention of the disease.

This study aims to assess the awareness of NMIBC patients referred to a tertiary reference center regarding the importance of smoking cessation, determine their access to smoking cessation programs and the effects of smoking cessation on recurrence rates of NMIBC.

## MATERIAL AND METHODS

### Study Design

Following the Institutional Review Board approval, data of patients who were under cystoscopy surveillance program (12) in our tertiary referral center were retrospectively collected. Patient demographics along with smoking habits were recorded. Only patients with pathologically confirmed bladder cancer and who were native Turkish speakers were enrolled. Patients who were referred to radical cystectomy or radiotherapy/chemotherapy because of muscle invasive disease or uncontrollable bleeding were not included. The data of cystoscopic surveillance of the patients together with the presence of recurrence disease were also recorded. The association of smoking habits and recurrence were evaluated.

### Data Acquisition

A urology resident applied an anonymous, 19-item, multiple-choice questionnaire upon arrival to the clinic for cystoscopic evaluation between November 2013 - March 2014. After obtaining patient demographics, the questionnaire collected data on employment history, socioeconomic status, monthly income, awareness of the association between bladder cancer and smoking and smoking status and history. Moreover, the patients were asked whether they received cessation advice from their urologists or other physicians or applied for a smoking cessation program.

Besides documentation of smoking habits of the patients, the recurrence pattern of the disease was also recorded. The recurrence rates of the smokers, ex-smokers and never-smokers were compared.

### Statistical analysis

Statistical analyses were performed using Number Cruncher Statistical System 2007 statistical software package program (NCSS, LLC, Kaysville, UT, USA). In addition to descriptive statistics (mean, standard deviation, median, interquartile range), data were-evaluated by independent t test for variables with normal distribution, by Fisher exact test and chi square test for comparison of qualitative data. Statistical significance was set as P <0.05.

## RESULTS

A total of 212 patients were invited to participate in the current study, 187 (88.2%) of which completed the survey and were included into the study. Table-1 summarizes the patient’s demographic characteristics and course of the disease.

**Table 1:** Demographic characteristics and course of the disease of patients who completed the survey and enrolled into the study.

Variable	Overall Patients (%)	Without Recurrence (%)	With Recurrence (%)	P

	n=187	n=103	n=84	
**Mean age (year)**	64.68±12.05	62.8±12.18	64.56±10.39	0.460
**Gender**				**0.316**
Female	20 (10.7)	18 (9.62)	2 (1.06)	
Male	167 (89.3)	85 (45.45)	82 (43.85)	
**Employment**				**0.354**
Working	69 (36.89)	37 (19.78)	32 (17.11)	
Retired	118 (63.10)	66 (35.29)	52 (27.80)	
**Education**				**0.173**
Illiterate	52 (27.8)	21 (11.22)	31 (16.57)	
Primary school	113 (60.4)	67 (35.82)	46 (24.59)	
High School	15 (8.0)	9 (4.81)	6 (3.20)	
College	7 (3.7)	6 (3.20)	1 (0.53)	
**Monthly Income**				**0.023**
<1000$	25 (13.36)	14 (7.48)	11 (5.88)	
1000-1500$	157 (83.95)	85 (45.45)	72 (38.50)	
>1500$	5 (2.67)	4 (2.113)	1 (0.53)	
Tumor grade*				**0.756**
Low Grade	112 (59.89)	77 (41.17)	35 (18.71)	
High Grade	75 (40.10)	26 (13.90)	49 (26.20)	
Tumor stage*				**0.420**
Ta	94 (50.26)	72 (38.50)	22 (11.76)	
T1	93 (49.73)	31 (17.11)	62 (22.15)	
**Intravesical therapy**				**0.046**
None	100 (53.47)	61 (32.62)	39 (20.85)	
Chemotherapy^§^	80 (42.78)	41 (21.92)	39 (20.85)	
BCG	7 (3.74)	1 (0.53)	6 (3.20)	

**BCG =** Bacillus-Calmette-Guérin

* Tumor grade and stage at time of diagnosis; ^§^ Epirubicin or Mitomycin-C

The smoking habits of the patients as well as their awareness on the possible causative factors of the bladder cancer are provided in Table-2. While 46.52% of the NMIBC patients were aware of the link between smoking and bladder cancer, 88 (47.05%) patients had no idea about the possible causes of their disease (Table-2).

**Table 2:** Smoking habits, awareness of the relationship between smoking and bladder cancer and their relationship with the recurrence of the disease.

Variable	Overall Patients (%)	Without Recurrence (%)	With Recurrence (%)	P

	n=187	n=103	n=84	
**Current smoking status**				**0.370**
Active smoker	48 (25.66)	18 (9.62)	30 (16.04)	
Ex-smoker	101 (54.01)	55 (29.41)	46 (24.59)	
Never smoked	38 (20.32)	30 (16.04)	8 (4.27)	
**Smoking status at time of diagnosis**				**0.460**
Active smoker	114 (61)	54 (28.87)	60 (32.08)	
Ex-smoker	35 (18.7)	19 (10.16)	16 (8.55)	
Never smoked	38 (20.3)	30 (16.04)	8 (4.27)	
**Awareness of the relationship between smoking and BC**				**0.316**
Yes	87 (46.52)	57 (30.48)	30 (16.04)	
Do not know	88 (47.05)	43 (22.99)	45 (24.06)	
No	12 (6.41)	3 (1.60)	9 (4.81)	
**Smoking is the major risk factor for BC**				**0.354**
Yes	76 (40.64)	41 (22.45)	35 (18.71)	
Do not know	97 (51.9)	57 (30.48)	40 (21.39)	
No	14 (7.48)	5 (2.67)	9 (4.81)	
Duration of smoking (years)	34.34±11.42	35.26±11.15	33.2±11.64	0.423
Cigarette packages smoked per day	1.21±0.76	1.3±0.76	1.16±0.74	0.428
**Smoking cessation attempts**				0.682
Yes	110 (58.82)	81 (43.31)	29 (15.50)	
No	39 (20.85)	24 (12.83)	15 (8.02)	
Duration since cessation smoking (years)	8.92±12.03	8.01±11.09	11.84±14.22	0.239
**Advised to quit smoking**				**0.420**
Yes	87 (46.52)	68 (36.36)	19 (10.16)	
No	62 (33.15)	35 (18.71)	27 (14.43)	
**Who advised to quit smoking**				**0.046**
Urologist	76 (40.64)	58 (31.01)	18 (9.62)	
Other physician	11 (5.85)	8 (4.27)	3 (1.60)	
Friends	8 (4.27)	7 (3.74)	1 (0.54)	
Internet/Social media	6 (3.20)	4 (2.13)	2 (1.06)	

**BC =** Bladder cancer

At the time of their diagnosis, 114 (61.0%) were active smokers, 35 (18.7%) were ex-smokers and 38 (20.3%) never smoked before. Of the actively smoking patients, 76.3% were advised to quit smoking and 66 (57.9%) of them quit smoking. Overall 76 (66.7%) actively smoking patients reported that their urologists advised them to stop smoking. Only 4.1% of the patients who were current or ex-smokers were referred to smoking cessation programs. Of these patients, a prescription medicine was recommended only to 5 (2.7%) patients to help stop smoking.

Unfortunately, 41 (62.12%) of the patients who quit smoking had started to smoke again.

After a mean follow-up of 32.28±11.42 months, 84 (44.91%) patients had recurrence. Patient’s smoking habits (p=0.370), awareness of the causative role of smoking in NMIBC (p=0.316) did not affect the recurrence (Table-2).

## DISCUSSION

Besides its causative role in development of various heart and lung diseases, it has also been confirmed that smoking increases the risk of recurrence and progression of NMIBC (13, 14). As the relationship between smoking and the course of the disease is well documented, the NMIBC is now accepted as a preventable condition (15, 16).

In an attempt to develop a model for predicting the outcomes of bladder cancer, Mitra et al. (17) combined molecular alterations involved in apoptosis, regulation of cell cycle, inflammation, angiogenesis and tumor invasion and smoking intensity. They showed that duration of smoking and number of cigarettes smoked daily were associated with COX-2 alterations and Bax expressions, respectively which in turn resulted in worse prognosis. Although previous reports demonstrated that the lowest risk of NMIBC is recorded in patients who never smoked before, cessation of smoking at time of diagnosis is also an important step in primary prevention of the disease (18, 19). Chen et al. evaluated the effects of cessation of smoking on the outcome of NMIBC using a post hoc questionnaire and interview (20). The 3-years recurrence free survival of continued smokers, non-smokers, ex smokers and patients who quit smoking within a year before and 3 months after the diagnosis was 45%, 57%, 62% and 70%, respectively. They also demonstrated that patients with bladder cancer who did not stop smoking had a 2.2-fold increased risk of disease recurrence when compared to patients who quit smoking (20). Recently, Al-Zalabani et al. conducted a systemic review of all meta-analyses, which evaluated the effects of modifiable risk factors on development of primary bladder cancer (21). Of the meta-analyses included, the one which has been performed by Van Osch et al. (13) reported the most comprehensive review regarding to smoking. The authors demonstrated that increasing duration since cessation of smoking reduced the development of bladder cancer, although former smokers had a 50% increased risk, even after more than 20 years of cessation. Moreover, Shiels et al. demonstrated that continuation of smoking might even result in development of a second malignant lesion in bladder (HR=3.67; 95% CI, 2.25 to 5.99) and kidney (HR=5.33; 95% CI, 2.55 to 11.1) among patients who survive from a previous smoking related cancer (7). Contrary to the previously published papers, our results failed to demonstrate any association between the recurrence rates and smoking cessation (p=0.370), a fact that can be explained by the low number of patients enrolled into the study. Furthermore, being a current smoker during major oncological surgeries had higher odds of overall, pulmonary, wound and septic complications compared with non-smokers (22). Although the risk persisted in former smokers, the odds of experiencing surgical complications were significantly lower than that of current smokers (22). Considering the high possibility of undergoing repetitive surgical operations, cessation of smoking also has an utmost importance in overall patient’s well being.

Nevertheless, almost half of the patients who were active smokers upon diagnosis of urothelial carcinoma did not quit smoking (23). In the current study, 42.1% of the patients who were smoking at time of diagnosis did not quit smoking. Besides highly addictive nature of nicotine, the patient’s limited awareness of the causative role of smoking in the development of bladder cancer may contribute to this finding. In a survey conducted among patients presenting to a urology clinic, only 36% of the sample reported that smoking was a risk factor for bladder cancer (24). In a similar prospective cross sectional study, Johnson et al. assessed the knowledge regarding the association between smoking and various diseases (25). Although 45.2% of the respondents were aware of the association between smoking and bladder cancer, this rate was much lower than that of respondents who were aware of the association between smoking and lung cancer (97.4%). Guzzo et al., on the other hand, reported greater awareness rates compared to previous studies (16). They demonstrated that despite having a bladder cancer, the ratio of the patients who were aware of the association between smoking and bladder cancer was lower than that lung cancer (86% and 100%, respectively). Authors explained these high rates for being a tertiary referral center. In our study population, only 46.52% of the patients recognize that smoking was a risk factor for bladder cancer while 40.64% addressed smoking as the major risk factor. Although the current study was also conducted in a tertiary referral center, the awareness of our patients was not as high as the previously published series, which may be explained with relatively worse educational and socioeconomic level of the study population.

Given the benefits of cessation of smoking on both the outcome of NMIBC and overall health of the patients, all physicians should counsel smokers to quit and offer cessation assistance. It has been shown that patients diagnosed with a cancer are much more motivated in smoking cessation when compared to smokers without a cancer (26). This effect is more pronounced within the first 3 months of the diagnosis of cancer (26). It seems that although urologists have the unique advantage of seeing their patients every 3 months during their cystoscopy controls, they do not handle this opportunity effectively. Within the actively smoking population of this study, 76.3% were advised to quit smoking and 66 (57.9%) of them quit smoking. Of these physicians, 66.7% were the urologists. We think that every urologist must pay more attention to establish patient awareness and should counsel their patients regarding cessation of smoking.

Although it has been demonstrated that a smoking cessation program including a strong physician message, behavior modification and medications may increase the smoking cessation rates from 5.4% to 21.7% (27), urologists also fail to refer their patients to smoking cessation programs. Guzzo et al. reported that only 10.4% of all the respondents in the study population were offered specific cessation aids by their urologist in particular (16). In our study, only 4.1% of the patients who were advised to quit smoking were referred to smoking cessation programs which were organized by the Ministry of Health and consisted of the combination of counseling and medications (nicotine patch, bupropion SR and varenicline) (28). In order to encourage and influence the urologists to engage in bladder cancer prevention and smoking cessation, The World Urologic Oncology Forum organized a global initiative (29). The results of this initiative may change the behavior of the urologists in the near future.

The study has several limitations. First of all, the study population was small and follow-up period was relatively short. The limited number of the patients and short follow-up period might be responsible for the inability of our results to demonstrate the beneficial effects of smoking cessation on recurrence rates of NMIBC. Second, our institute is a tertiary referral center and thus may not reflect the overall NMIBC population. Most of our patients come from rural parts of the country with lower socioeconomic status. Also, the rate of illiterates is relatively higher than that of the whole country, a limitation that may affect the accuracy of the responses of the patients. Considering the potential risk of recall bias due to the low socioeconomic status of our patients, we did not collect data regarding the exact time interval between the surgical procedure and the recurrence of the disease. Therefore, we could not perform Cox proportional hazards, which is a statistical method for assessing the impact of variables upon the time a specified event takes to happen. Another limitation of the study is the administration of a semi-structured interview, which bears a risk of obtaining misleading information from the respondents. However, such a risk exists in all kinds of interviews regardless of being structured or non-structured. In order to overcome this limitation, the questions in the survey were carefully selected not only to cover but also to secure the main objective of the study. In the current study, the questionnaires were conducted by the help of urology residents in order to prevent any misunderstandings. Also, a neutral and non-offensive language was used to avoid biased answers. Finally, the retrospectively collected data relies on self-reported questionnaires that were not validated yet. In spite of these limitations, the awareness of NMIBC patients regarding the importance of smoking cessation has not been well studied in a developing country thus our results may be beneficial for health policy makers and planners.

## CONCLUSIONS

The majority of the NMIBC patients were not aware of the association between smoking and bladder cancer in our study group. Although most of the physicians advised to quit smoking, significant amount of the patients were still active smokers during follow-up. Only a small proportion of the patients were referred to smoking cessation programs. Urologists must take a more active role in the battle against smoking and refer those patients to smoking cessation programs as needed. On the other hand, larger study populations with longer follow-up periods are needed to demonstrate the beneficial effects of smoking cessation on recurrence rates.
